# Smartphone based colorimetric point-of-care sensor for abused drugs: case of baclofen determination in urine

**DOI:** 10.1186/s13065-023-01093-z

**Published:** 2023-12-10

**Authors:** Mariam O. Abd el-Aziz, Ahmed H. Nadim, Hany H. Monir, M. Nebsen, Sameh E. Younis

**Affiliations:** 1 Department of Pharmaceutical Chemistry, Faculty of Pharmacy, Pharos University in Alexandria, Alexandria, Egypt; 2 Analytical Chemistry Department, Faculty of Pharmacy, Cairo University, Cairo, Egypt

**Keywords:** Smartphone, POCT, Baclofen, Drug abuse, Therapeutic drug monitoring, Colorimetric reaction

## Abstract

As a GABA-β receptor agonist, the central muscle relaxant Baclofen (BAC) has a potential of abuse. Unfortunately, the sense of wellbeing and pleasure is obtained at very high BAC doses. This is associated with many life-threating or even fatal cases due to neurological and respiratory failures. Moreover, having narrow therapeutic index makes BAC a high-risk drug. This is potentiated in case of long-treatment regimen or off-label use in smoking and alcohol cessation protocols. Until now, there is no rapid diagnostic test available for BAC screening. Therefore; It is quite difficult to routinely monitor cases on BAC regimen. On the other hand, smartphone-based colorimetric point of care testing (POCT) is displacing conventional analytical approaches in the detection and assay of abused drugs as well as therapeutic drug monitoring. It offers on-site, rapid, easy, affordable and interpretable analysis. Incorporating smartphone as a portable device facilitates its application, especially in remote areas and low-income countries. For the first time, the current work presents a smartphone-based colorimetric POCT for BAC analysis in urine without interference from urine matrix. It depends on BAC reaction with naphthoquinone sulfonate (NQS) in highly alkaline aqueous medium. The developed color was captured in a customized photo box using smartphone camera. Then, intensity of the blue channel was measured by a software application “Color Analyzer”. All parameters were optimized with respect to the colorimetric reaction, photographing and smartphone-based analysis. All parameters were successfully investigated according to FDA guidelines for bioanalytical method validation. Also, all POCT criteria were considered as per WHO requirements. This method could determine BAC, linearly, from 0.02 to 0.21 mmol L^−1^ in urine. Moreover, LLOQ was lower than the expected BAC therapeutic concentrations in urine. The proposed method proved high reliability and suitability to analyze BAC in urine. This strongly recommends its routine application in screening BAC abusers and BAC therapeutic monitoring.

**Graphical Abstract:**

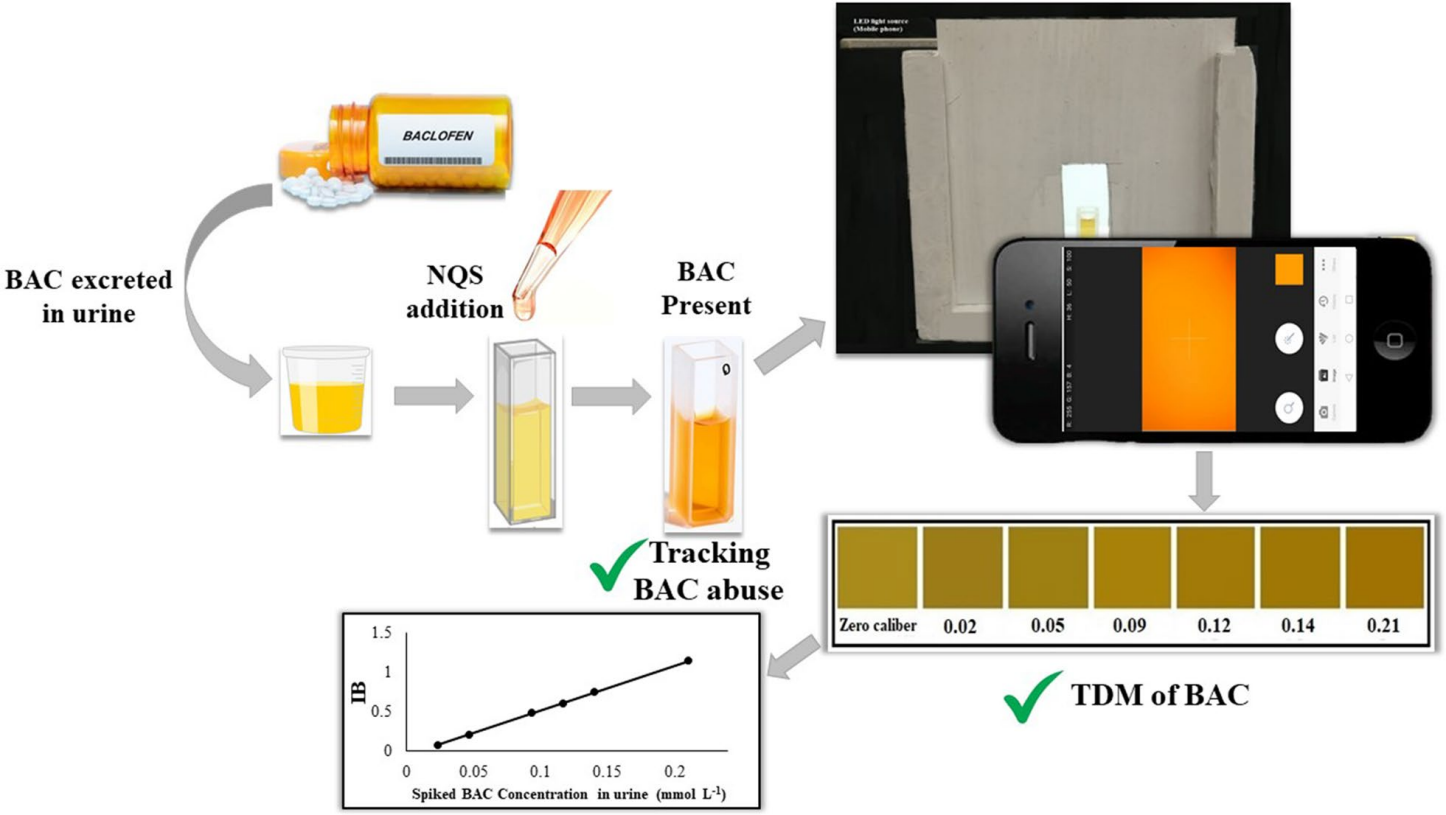

## Introduction

Baclofen (BAC) is chemically named as 4-amino-3-(4-chlorophenyl) butanoic acid [1]. It functions by stimulating GABA-β receptors at both pre and post synaptic neurons, which leads to muscles’ relaxation, in addition to relieving anxiety symptoms [2, 3]. BAC has been approved by FDA as a skeletal muscle relaxant for treating severe spasticity and concomitant pain accompanying serious illnesses as; Multiple sclerosis and spinal cord lesions [4]. Additionally, it has an off label use for treating alcohol use disorders, together with relieving alcohol dependence related anxiety [5, 6]. It was found that orally administered BAC has 70 to 85% bioavailability, with almost 75% of the administered dose being excreted unchanged in urine [7, 8].

BAC is believed to have a potential of being abused, due to its direct effects on neurotransmitters of CNS, together with affecting the mesolimbic reward system in the brain. This offers sedative effects to addicts. Unfortunately, such physiological and psychological effects are attained only when BAC is being overdosed [9]. Studies claimed that doses of 180 mg and higher of BAC were taken by addicts, which are very high compared to BAC therapeutic doses (10 and 25 mg) [10]. Some reports stated that overdosing of BAC is fatal due to the neurological toxicity and respiratory failure [10–12]. As a consequence, a simple and readily available detection test is necessary to rapidly screen BAC abusers. Moreover, a regular therapeutic monitoring is recommended to control unintentional overdosing of BAC in case of long term therapy.

A thorough literature survey reveals plenty of adopted methods for BAC determination in pharmaceutical formulations and biological fluids [13]. Among of them are; UV spectroscopy [14–18], spectrofluorimetry [19–21], capillary electrophoresis [20], RP-HPLC [22], HPTLC [7, 23], LC–MS/MS [24, 25], and potentiometry [26]. However, only one method that depended on microfluidics-based fluorimetric technology was reported for BAC [27].

On the other hand, point of care testing technology (POCT) are based on the same principles as those of conventional instrumental-based analytical approaches in using colorimetric, fluorescent or electroanalytical methods in detection. However, POCTs differ in applying these methods on portable simple devices. They offer several significant advantages over conventional instrumental techniques. POCTs are readily available onsite, affordable and offering rapid test results. These merits make them more convenient to be adopted in remote areas, especially in the developing countries. In addition, they require neither sophisticated infrastructure nor trained personnel. As a result, POCTs are now gaining world’s attention to substitute laboratory-based approaches, whenever possible. This state of the art technology became a golden tool in diagnosis and quantitative analysis of abused drugs. Also, it facilitated the therapeutic monitoring of narrow therapeutic index drugs; allowing rapid and effective medical interventions [28, 29].

Among of the applied analytical methods in POCTs; colorimetry, which is commonly adopted as detection technique. It achieves the main target of this technology through utilizing readily available, cheap and easily handled chromogenic reagents, together with the ease of interpreting the obtained test results. Moreover, it can analyze molecules in various samples’ matrices either in their dosage forms or in biological fluids like saliva, plasma and urine [29–33]. In addition to qualitative screening of targeted molecules, a semi-quantitative assay can be achieved by the aid of already established calibration charts. Even, quantitative approach can be attained through smartphone-based applications [34–37].

Smartphones empower the perspective of substituting the laboratory-based analytical approaches by the POCTs. The main driving factors are the already integrated powerful cameras and the free-to-use IOS and android applications. So, in fast and simple few steps, one can detect target molecules through taking pictures by smartphones integrated cameras. They are sensitive enough to observe minute color differences. And by the aid of installed application, pictures can be simultaneously quantified by processing the obtained color values from different color scales; RGB, CMYK, HSV and others. The color scale that gives more reliable and specified results corresponding to the analyzed class of molecules was chosen for analysis [31]. A deduction can be driven here that smartphone-based colorimetric POCTs will not only be an alternative to conventional analytical approaches, but they will also be the ideal next generation of reliable POCTs.

The literature review showed some smartphone-based colorimetric POCTs that are developed for screening of abused drugs [31, 38, 39], detection of adulterated drugs [40] and quantitation of toxic molecules that may be found in environment or in pharmaceutical preparations [36, 41, 42].

The current research was targeted mainly to develop and optimize an analytical method that can be used alternatively to the conventional laboratory-based methods in detecting and quantitating BAC in urine. The proposed method would provide a rapid, simple and onsite assay. It applied a colorimetric POCT using a smartphone camera with a preinstalled RGB software. This would facilitate spotting BAC abusing cases, in addition to monitoring of cases on BAC regimen to allow quicker medical interventions in case of overdosing and subsequent toxicity.

## Materials and methods

### Chemicals and reagents

Baclofen (BAC) of 99.8% purity was obtained from Pharaonia Pharmaceuticals Co., Alexandria, Egypt. 1,2-naphthoquinone-4-sulfonate (NQS) of 97% purity was purchased from Aldrich Chemical Co., USA. Analytical grade acetonitrile, methanol, ethanol, sodium hydroxide, acetic, boric and phosphoric acids were purchased from El-Nasr Chemical Co., Alexandria, Egypt. Freshly prepared distilled water was used for all experiments.

### Apparatus and software

The photographing process was performed using Huawei Y9 smartphone with a built in camera. The rear camera used for detection, consists of a 13 MP and 2 MP dual depth sensor lenses, with lens aperture of f/1.8 and f/2.4, respectively. Its sensor operates in 4:3 aspect ratio with an image resolution of 4128 × 3096 pixels. It is featured with the AI technology which recognizes the finest details of the pictures. A 16 dual LED white flashlight of another mobile phone was used as an illumination source to provide a consistent incident light above the sample cuvettes. A customized wooden made photo box (15 cm L × 15 cm W × 15 cm H) was built to capture images of the colored solutions, to omit the effects of any other interfering lights coming from the surrounding environment. An inlet (7.5 cm H × 2 cm W) was opened in the box’s front side, for the smartphone camera. Two boxes were designed; Each of different background color (White and black) for optimization purposes of the developed method. The ready for measuring colored solutions were placed in rectangular glass cuvettes, to facilitate the process of photographing [36]. The processing of captured images took place by the use of an android software named “Color Analyzer” that was previously installed from “Google play store” on the smartphone being used in analysis. All prior optimization trials of the adopted colorimetric reaction were carried out using UV-1800 thermospectronic Helios Alpha (UK) UV–VIS spectrophotometer, Shimadzu Corporation, Japan, connected to Harvest computer system. Quartz cells of 1 cm were employed. pH measurements were recorded using digital pH meter 3310 Jenway.

### Preparation of reagent and buffer solutions

0.25% w/v aqueous solution of NQS was prepared by dissolving 250 mg reagent powder in 100 mL distilled water [43]. Britton Robinson buffer (BR) used in optimization studies of the pH was prepared from equal volumes of 0.1 mol L^−1^ solutions of acetic acid, boric acid and phosphoric acid, each in 100-mL volumetric flask, then the desired pH range (3.0–11) was acquired using certain volumes of 0.1 mol L^−1^ NaOH solution [44].

### Preparation of standard solutions

Standard stock solution of BAC (4.68 mmol L^−1^) was prepared using methanol as a solvent and stored under refrigeration. Subsequent dilutions with distilled water were made to prepare adequate aqueous working solutions.

### Construction of calibration curve

#### Preparation of human urine samples

Pooled urine samples were collected from ten adult males in their 30’s. They had normal kidney functions and were not suffering from any medical conditions before samples collection. 1.0 mL aliquots of the pooled blank urine were transferred into a series of test tubes. Each sample was spiked with BAC in the concentration range of 0.02–0.21 mmol L^−1^ urine, each concentration was prepared in triplicates. They were separately diluted with distilled water by a factor of (1:0.5 v/v) for samples and water, respectively. To each diluted sample; 3.0 mL of acetonitrile were added and centrifugation was carried out at 4000 rpm for 15 min. Then, 3.0 mL of the supernatant were transferred to a 10-mL volumetric flask for subsequent colorimetric reaction.

#### Colorimetric reaction

To each processed urine sample; 2.0 mL of 0.5 mol L^−1^ NaOH solution and 1.0 mL of 0.25% w/v NQS reagent solution were added. The solutions were mixed well, left to stand at room temperature for 30 min, and then, completed quantitatively with distilled water to 10 mL.

#### Photographing and quantitative processing of the captured images by smartphone

**Fig. 1:**
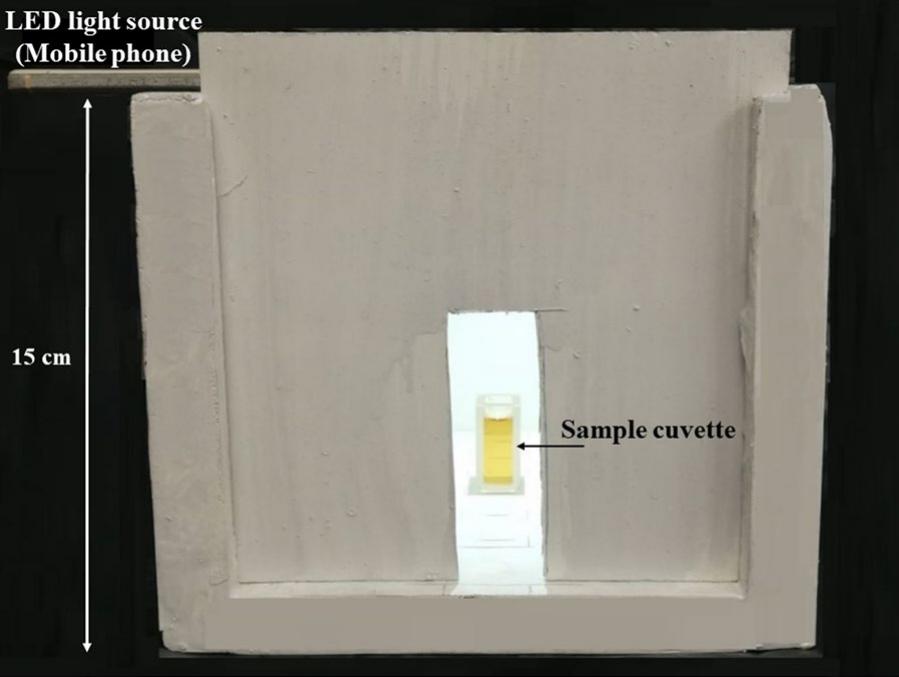
Miniaturized photo box with sample cuvette placed in a horizontal distance of 7.0 cm from the smartphone camera

From each solution; 1.0 ml of the readily formed colored solution was transferred to a rectangular glass cuvette. Then, the sample cuvette was placed inside the miniaturized photo box in a horizontal distance of 7.0 cm from the smartphone camera which is settled at the box inlet as shown in Fig. 1. In addition, the LED flashlight that is fixed in a vertical position from the cuvette was turned on to allow the incident light to go through the solution. Then, the image was captured through “Color Analyzer” application, against the box’s white background, by focusing the camera lens on the center point of the sample cuvette.

The blue channel value of the RGB scale corresponding to each spiked concentration was recorded. Then, the relative color intensity was derived from the equation **(**${\varvec{I}}{\varvec{B}}={\varvec{L}}{\varvec{o}}{\varvec{g}}\boldsymbol{ }\frac{{\varvec{B}}^\circ }{{\varvec{B}}{\varvec{s}}}$**)** [36].where; IB is intensity of the blue color value, B° and Bs are the initial blue color values of the blank and sample, respectively.

The calculated relative intensities were plotted against the corresponding spiked BAC concentrations (mmol L^−1^) to construct the calibration curve.

### Preparation of quality controls (QCs)

Quality control samples were prepared by spiking blank urine samples with working standard solutions of BAC at five concentration levels representing LLOQ, LQC, MQC, HQC, and ULOQ (0.02, 0.07, 0.12, 0.14 and 0.21 mmol L^−1^ urine, respectively) as required by FDA guidelines for validation of bioanalytical methods [45]. Then, the proposed method was applied as formerly described in construction of calibration curve ("Construction of calibration curve" section).

## Results and discussion

### Spectral characteristics

**Fig. 2:**
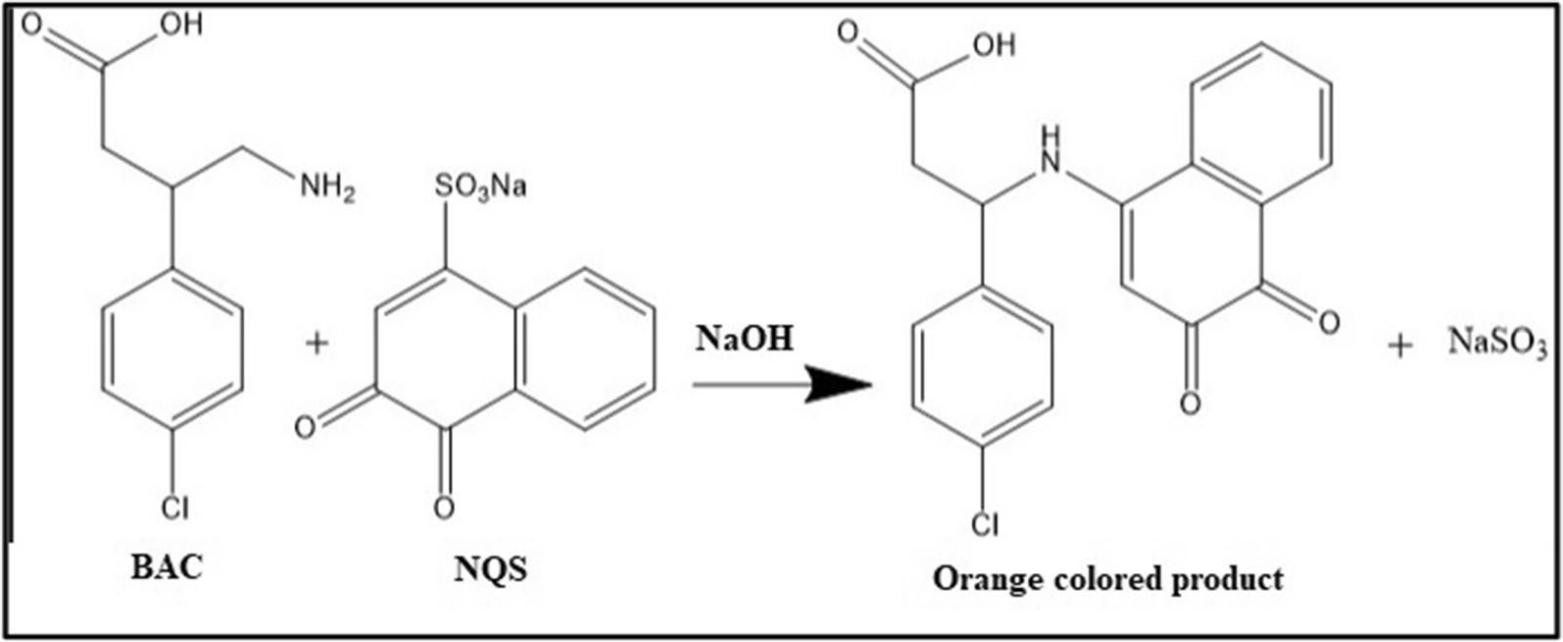
Proposed mechanism of nucleophilic substitution reaction of BAC with NQS in alkaline medium

The proposed POCT method is based on a colorimetric reaction of BAC using the derivatizing reagent; NQS. It reacts with the aliphatic primary amine moiety [43] exists in BAC molecule. As shown in Fig. 2, a nucleophilic substitution reaction between the aliphatic (-NH_2_) moiety of BAC and the sulfonate position of NQS molecule occurs in alkaline pH, yielding a stable orange to red colored product which absorbs at λ _max_ of 470 nm.

### Optimization of the colorimetric reaction

**Fig. 3:**
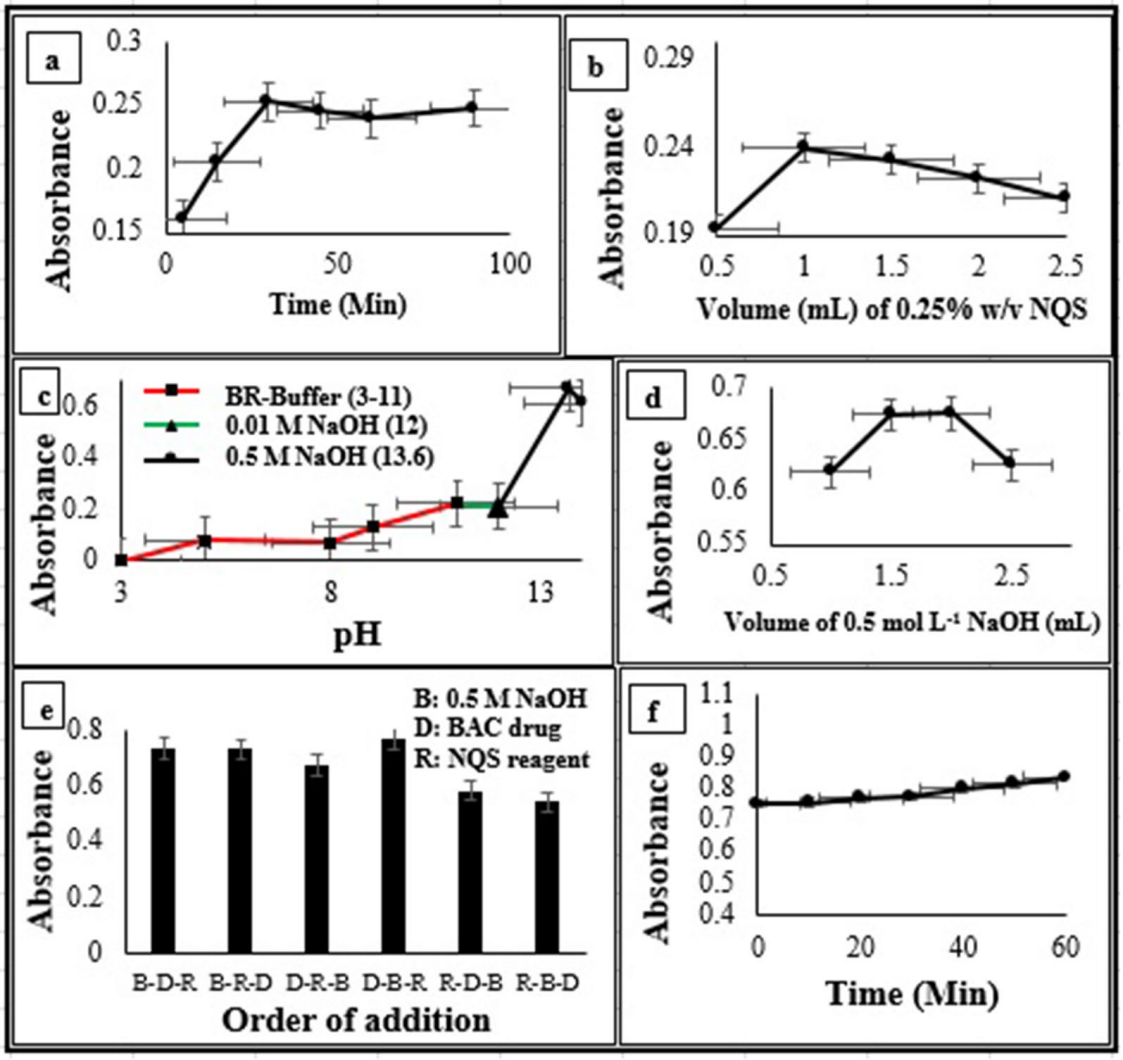
Effect of colorimetric reaction parameters on the product absorbance of 0.14 mmol L^−1^ BAC at 470 nm: **a** Reaction time, **b** Reagent volume, **c** Medium pH, **d** pH modifier volume, **e** Order of addition and **f** Stability of the colored solution ready for measurement

Various parameters were studied and optimized. The pH of the reaction medium; BR buffer was used to adjust pH from 3 to 11, while 0.01–0.5 mol L^−1^ of NaOH solutions were used to attain higher pH ranges from 12–13.6. Concentration of the reagent solution was studied using 0.5–2.5 mL of 0.25% w/v NQS solution. Time required for complete reaction was observed over time period starting from 5.0–90 min. Order of addition of reaction components and stability of the formed colored product ready for measurement. Optimization studies were held in room temperature using BAC solution at concentration level of 0.14 mmol L^−1^ and the resulted orange color was measured at λ _max_ of 470 nm using UV–Vis spectrophotometer. The preliminary studies declared that 30 min were sufficient to reach the maximum reaction sensitivity as shown in Fig. 3a. However, it was observed that the characteristic orange colored product started to appear after 5.0 min of the reaction. Thus, BAC presence can be quickly screened after only 5.0 min of the reaction. A volume of 1.0 mL of 0.25% w/v NQS aqueous solution was required to obtain maximum color intensity as revealed in Fig. 3b. Different pH modifiers were tried. The maximum color intensity was achieved at highly alkaline medium, through adding 2.0 mL of 0.5 mol L^−1^ NaOH (pH = 13.6), Fig. 3c, d. Data of studies of the order by which reaction components were added to obtain the maximum response are demonstrated in Fig. 3e. The drug had to be added first, followed by adding the pH modifier (NaOH) solution. Then, NQS solution was added. The resulted colored solution remained almost constant for one hour at room temperature, Fig. 3f.

### Optimization of smartphone-based analysis

In all experiments; Samples’ cuvettes were placed in photo box and then photographed by smartphone camera. The use of the photo box was found essential to control the surrounding light that may affect the intensity of the captured color by the camera lens. It allowed only the incident light from the LED flash light (16 W) to pass through sample cuvette. Thus, a fixed light intensity was being adopted throughout all measurements. Moreover, rectangular glass cuvettes were adopted as the sample cuvettes. Their flat surface allowed more consistent light distribution. Hence, more accurate results were recorded by the smartphone camera.

The box was made of wood due to its robustness against environmental conditions. It was customized to have dimensions of (15 cm L × 15 cm W × 15 cm H). In addition to ease of photographing, such dimensions made sample position to camera lens as well as light source suitable to get reliable photographing of colors.

Capturing high quality images can clearly show the minute color variations in response to concentration changes of the target drug. This ensures high sensitivity and reliability of the proposed method. Various factors associated with the photographing process of the developed colored solutions, together with others that affect the analysis of those captured images are studied and optimized. These factors include; Color scale used in analysis, quality of the imaging background and distance at which the camera has to be settled from sample cuvette.

#### Color scale

The nature of the colored solution determines the most suitable color model to describe it. The RGB color scale provided by “Color Analyzer” application was found the optimum scale to record the color variations developed for different BAC concentrations. The optimum color channel (Red, green or blue) from RGB color scale was deduced by studying the sensitivity, together with correlation between the obtained color intensity of each color channel in response to different BAC concentrations (0.02–0.21 mmol L^−1^). As per Table 1a, the blue channel offered significantly higher linear relationship (r^2^) and sensitivity (S) between BAC concentrations and the developed colors. While, limited r^2^ and S were obtained for red and green channels. So, calculated intensities of the blue channel (IB) were recommended for further BAC analysis.

**Table 1:** Optimization of parameters affecting smartphone based colorimetric analysis: a) Color scale channel and imaging background and b) distance between smartphone camera and sample cuvette

a			
		S^a^	r^2a^
RGB scale channel	Red	0.292	0.7274
	Green	0.874	0.9780
	Blue	11.52	0.9989
Imaging background	White	11.52	0.9989
	Black	4.389	0.9968

b					
Distance (cm)	3.0	5.0	7.0	9.0	11.0
RSD % ^b^	14.624	7.003	3.915	7.049	3.972

^**a**^Sensitivity (Slope) and r^2^ values were calculated using BAC concentrations of 0.02–0.21 mmol L^−1^

^**b**^RSD% values were calculated from mean of three measurements (n = 3)

#### Imaging background

IB signals of series of BAC concentrations (0.02–0.21 mmol L^−1^) were recorded, through photographing the colored solutions against two monochromatic white and black backgrounds. The white background demonstrated clearer images with higher quality than those of the black one. This was observed in terms of sensitivity and linearity between IB of photographed colored solutions and corresponding BAC concentrations (Table 1a). Thus, white background was employed for the proposed method.

#### Distance from smartphone camera to sample cuvette

The distance at which images of the colored solutions were captured was found to have a significant impact on the repeatability of the measurements. So, IB signals of three replicates of 0.09 mmol L^−1^ of BAC solution were recorded from images captured at varying distances (3–11 cm). Capturing images at a distance of 7.0 cm, between sample cuvette and camera lens fixed in horizontal position, allowed the best camera lens focus and thus more reproducible results were obtained as revealed by Table 1b.

### Analysis of BAC in spiked urine samples by the proposed smartphone-based colorimetric POCT method

**Fig. 4:**
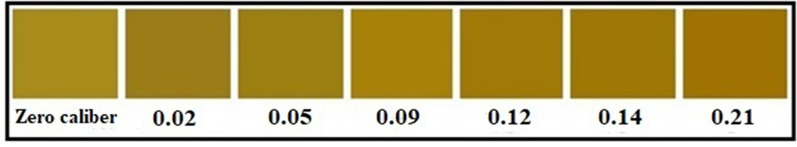
Color key chart representing different BAC concentrations (mmol L^−1^) spiked in urine

The applicability of the proposed smartphone-based colorimetric POCT method was checked for BAC screening and quantitation in urine samples. Protein precipitation is a simple and easy extraction method which is suitable for the concept of POCT. Thus, it was adopted for BAC extraction from urine matrix. Acetonitrile, methanol and ethanol (in different ratios to urine sample volume) were tried as precipitating solvents. However, the best recovery was obtained using acetonitrile in a ratio to urine sample of 3:1 v/v. A series of urine samples spiked with BAC in concentration range of 0.02–0.21 mmol L^−1^ urine were analyzed by the proposed method. The color differences between the produced orange colored solutions corresponding to each spiked BAC concentration can be visualized clearly by the naked eye. Therefore, a color key chart was developed as shown in Fig. 4. This allowed rapid as well as simple screening of BAC presence, together with the ability to assay BAC semi-quantitatively in the tested urine samples.

Moreover, the smartphone-based approach was converted to be a mini-spectrophotometer to analyze samples quantitatively. The incorporation of smartphone camera with the aid of ‘Color Analyzer’ software enabled a reliable and accurate estimation of BAC concentrations in urine samples. BAC spiked concentrations were predicted from the intensity of the color value recorded from blue channel of the RGB scale (IB).

### Validation

The performance of the proposed method for BAC determination in urine samples was validated as assigned by FDA guidelines for validation of bioanalytical methods [45]. Also, the performance of the proposed method to be considered as a POCT method was evaluated as per WHO criteria for point of care devices [38].

#### Calibration curve, lower limit of quantitation (LLOQ) and sensitivity

**Fig. 5:**
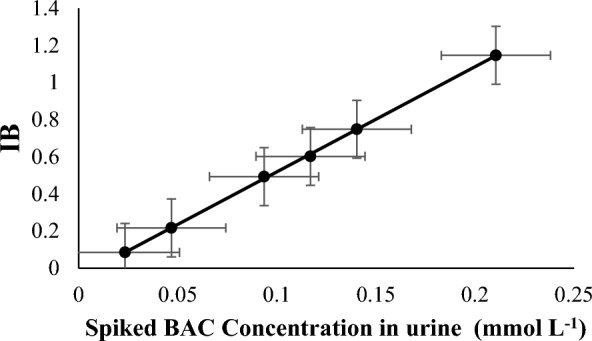
Calibration curve of the proposed smartphone based POCT method

To construct the calibration curve; IB signals of six non-zero calibrators of BAC spiked in urine samples at concentration levels of 0.02, 0.05, 0.09, 0.12, 0.14 and 0.21 mmol L^−1^ urine, were plotted against the corresponding BAC concentrations **(**Fig. 5**)**. Regression analysis was carried out on calibration data using the least squares method. Various linearity parameters were calculated; Determination coefficient (r^2^), slope (b), intercept (a), standard deviation of slope (S_b_), standard deviation of intercept (S_a_), F value and significance F. All calculated parameters were found to be acceptable **(**Table 2**)**. The good linearity of the proposed method was confirmed by high determination coefficient (r^2^ > 0.99), together with low standard deviation of intercept (%S_b_ < 2%) and high F value. Also, the small value of significance F indicated low scattering points around the regression line.

**Table 2:** Regression parameters of the proposed smartphone-based colorimetric POCT method for BAC determination in urine

Parameter	BAC
Linearity range (mmol L^−1^ urine)	0.02–0.21
LLOQ (mmol L^−1^ urine)	0.02
Determination coefficient (r^2^)	0.9996
Slope ± S_b_	5.66 ± 0.055
Intercept ± S_a_	− 0.047 ± 0.007
S_b_%	0.977
S _y/x_	0.007
F value	10,476.36
Significance F	5.463 × 10^–8^

Back calculated concentrations of the non-zero calibrators were found to be within ± 20% for LQC, MQC and HQC and within ± 25% for both LLOQ and ULOQ of the theoretical concentrations. This further assured the reliability of the proposed method.

Moreover, LLOQ; Lowest non-zero calibrator covered by the calibration curve [45], was 0.02 mmol L^−1^ BAC in urine. It is lower than the expected therapeutic concentrations of BAC excreted in urine (0.03 mmol L^−1^ and 0.06 mmol L^−1^) following single oral administration of the available 10 and 25 mg tablets, respectively [46]. This ensured the suitability of the proposed method in assaying real BAC containing samples.

Furthermore, the sensitivity of the method was confirmed by measuring responses from four replicates at LLOQ level (0.02 mmol L^−1^ BAC in urine). Recovery%, RSD% and total error were calculated and found to be 106.11% (± 25%), 14.05% (± 25%) and 20.16% (< 40%), respectively. Thus, sensitivity of the proposed method was proved for determining BAC excreted in urine.

#### Accuracy and precision

The intra-day as well as inter-day accuracy and precision of the obtained responses were evaluated. The previously described method was applied to the five QCs (0.02, 0.07, 0.12, 0.14 and 0.21 mmol L^−1^), each in four replicates. Recovery %, RSD % and total error were calculated for each QC level. As shown in Table 3, recovery % and RSD % values were all within the acceptable limits of ± 20% (± 25% for LLOQ and ULOQ levels). Also, total error results for QCs were within ± 30% (± 40% for LLOQ and ULOQ). This indicated the high accuracy and precision, and by consequence the reliability of the proposed method in determining BAC in urine samples.

**Table 3:** Accuracy and precision of the proposed smartphone-based POCT method for BAC determination in urine (n = 3)

Spiked BAC concentration^*^	Intra-day			Inter-day		
	Recovery %	RSD %	Total error	Recovery %	RSD %	Total error
0.02	109.96	12.78	22.74	112.36	11.31	23.67
0.07	92.98	4.11	11.13	108.15	11.09	19.24
0.12	98.44	7.83	9.39	102.42	12.66	15.08
0.14	96.72	4.85	8.13	104.10	5.52	9.62
0.21	95.14	7.32	12.18	108.48	10.97	19.45

*Concentration in mmol L^−1^ urine

#### Selectivity

The selectivity of the proposed method towards the targeted BAC was checked in two ways. First, the potential interference of the urine sample matrix was checked. Blank urine samples from ten individual sources were analyzed. Almost 90% of the un-spiked samples resulted in responses that were below quality level of BAC at its LLOQ level. This conformed with the acceptance criteria that ≥ 80% of un-spiked samples should be BQL. Second, for further selectivity evaluation; LLOQ and HQL spiked urine samples were analyzed in triplicates. The obtained recoveries % at both levels were within ± 20% and ± 25% for HQL and LLOQ, respectively **(**Table 4**)**. Consequently, the ability of the proposed method to detect and quantify BAC without potential interference from the urine matrix was confirmed.

**Table 4:** Evaluation of selectivity of the proposed smartphone-based colorimetric POCT method for BAC in urine (n = 3)

Spiked BAC concentration^*^	Recovery%	RSD%
0.02	104.88	6.55
0.14	100.78	8.99

* Concentration in mmol L^−1^ urine

#### Recovery

Urine samples spiked with BAC at LQL, MQL and HQL were analyzed. Their responses were compared with urine samples spiked with similar BAC concentrations after extraction. The calculated overall mean recovery was 91.20% **(**Table 5**)**. This proved mean value and its steady magnitude added a lot to the method’s reliability.

**Table 5:** Recovery study of the proposed smartphone-based colorimetric POCT method for BAC in urine

Spiked BAC concentration^*^	Recovery%	RSD%
0.07	89.59	4.88
0.12	92.39	7.97
0.14	91.60	4.96

*Concentration in mmol L^−1^ urine

#### Stability

The stability of BAC in urine matrix was determined by studying its bench top, extract, freeze–thaw, long term and stock solution stability approaches. All stability studies were held in triplicates on LQL and HQL samples (0.07 and 0.14 mmol L^−1^ urine, respectively). Bench top and extract stability studies were held by leaving the pre and post extracted QCs, respectively, for six hours at room temperature. Freeze–thaw stability was tested by exposing the pre-extracted QCs to three cycles of freeze and thaw, with a minimum of 12 h between cycles. In long term stability, pre-extracted QCs were assessed after being stored in -20 °C for 30 days; A period from the date of initial samples collection to the date of the last sample analysis. Additionally, stability of BAC stock solution was checked by its storage at 4 °C and 25 °C for 30 and 6 days, respectively. The obtained recoveries% were compared with those obtained from freshly prepared QCs. As per Table 6, excellent stability was proved for BAC in the urine matrix during storage, processing and analysis phases.

**Table 6:** Evaluation of BAC stability in urine by applying the proposed smartphone-based colorimetric POCT method (n = 3)

Stability approach	Recovery%	
	0.07 mmol L^−1^ BAC in urine	0.14 mmol L^−1^ BAC in urine
Bench-top	111.96	111.88
Extract	87.58	83.51
Freeze–thaw	104.42	97.38
Long term	108.13	104.59
Stock solution 4 °C 25 °C	104.42 113.93	102.27 112.23

#### Dilution factor

QCs at the concentration levels (0.23, 0.26 and 0.28 mmol L^−1^) were prepared. They were analyzed in five replicates using the planned dilution factor (1:0.5 v/v) for urine and water, respectively. The highest values of Er % and RSD % were 14.97% and 17.53%, respectively. They were within the acceptable limits (± 20%). In addition, no prosone effect was observed at any QC level. Thus, the applied dilution factor proved high reliability to analyze BAC at the high concentrations, excreted in urine in toxicity cases.

#### Validation of the proposed POCT method as per WHO criteria

The capability of the proposed method to be considered as a point of care testing method should be assessed. Some parameters were assigned by WHO organization in the term “ASSURED” have to be evaluated. In other words; The proposed POCT method should be affordable, sensitive, selective, user friendly, rapid, equipment free and deliverable to end users. The proposed method was found to be **affordable.** It only requires the use of low-cost colorimetric reagent with the aid of camera of smartphone that is believed to be readily available with end users. Regarding **sensitivity,** the proposed method can detect BAC concentration as low as 0.02 mmol L^−1^ urine, which is lower than the therapeutic BAC concentrations that are expected to be present in urine. Moreover, the method can detect low BAC concentrations without experiencing any interfering effect from the urine matrix, proving the required **selectivity**. Being **user friendly** is achieved as the method involves neither sophisticated instruments, nor multiple steps, so it can be operated comfortably by untrained personnel. Also, **rapid** instantaneous prediction of test results can be accomplished once the images of the produced colored solutions were captured by the smartphone camera. However, for quantitation purposes, it is recommended to leave the colorimetric reaction for 30 min to proceed before capturing the images to obtain representative responses. In addition, the method is **equipment free**, no specific equipment is needed to perform the proposed method. Only a camera and a LED flashlight of smartphone is required. As a **deliverable** method, it is applied using portable tools that can be easily available on the spot. As a conclusion, the developed smartphone-based colorimetric POCT method was confirmed as a reliable and readily accessible method to be adopted in assaying BAC in urine samples. Also, it can be operated feasibly in remote areas and developing countries.

In addition, the proposed colorimetric method had better analytical performance compared to the previously reported colorimetric literature regarding linearity, LOD, accuracy and precision (Table 7).

**Table 7:** Comparison of colorimetric methods for BAC determination

Chromogen	Linearity (mmol L^−1^)	Accuracy (Recovery%)	Precision (RSD%)	Sample	Determination method	Refs
NQS	0.02–0.21	98.65	7.38	urine	Smartphone based POCT	This work
Ninhydrin	1.87–6.55	Not considered in details		Pharmaceutical dosage form	Conventional spectrophotometry	[16]
4-chloro-7-nitro-2,1,3-benzoxadiazole	0.02–0.33	Not evaluated		Pharmaceutical dosage form	Conventional spectrophotometry	[14]
Iron (III)/ potassium hexacyano ferrate	0.05–23.4	Not evaluated	0.71	Pharmaceutical dosage form	Flow injection spectrophotometry	[17]
Vanillin Eosin Acetylacetone/ formaldehyde	0.05–0.16 0.02–0.09 0.02–0.12	100.29 101.00 99.49	1.02 1.22 0.70	Pharmaceutical dosage form	Conventional spectrophotometry	[18]

## Conclusion

The proposed method represents the first attempt to employ smartphone-based colorimetric POCT approach for abuse detection and therapeutic monitoring of BAC. It utilizes a simple available reagent in a fast procedure which is accomplished at the place of potential abuser or patient care. The method proved high accuracy and precision for BAC detection and quantification in urine. Adopting smartphone in analysis excludes the need for any specific analytical instrument, even the portable ones. In positive way, this reflects on the method suitability for distant locations and developing countries. The sensitivity, selectivity and simplicity of the suggested method were high enough to recommend substituting the laboratory-based analytical methods for BAC analysis in urine.

## Data Availability

All data generated or analyzed during this study are included in this published article.

## Abbreviations

BAC: Baclofen
BR buffer: Britton-Robinson buffer
HQC: High quality control
IB: Intensity of blue color
LED: Light emitting diode
LLOQ: Lower limit of quantitation
LQC: Low quality control
QCs: Quality controls
MQC: Mid quality control
MP: Mega pixel
NQS: 1,2-Naphthoquinone-4-sulfonate
POCT: Point-of-care testing
POCTs: Point of care tests
RGB scale: Red–Green–Blue scale
RSD: Relative standard deviation
ULOQ: Upper limit of quantitation
